# Developmental and Reproductive Effects of SE5-OH: An Equol-Rich Soy-Based Ingredient

**DOI:** 10.1155/2009/307618

**Published:** 2008-12-15

**Authors:** Ray A. Matulka, Ikuo Matsuura, Tohru Uesugi, Tomomi Ueno, George Burdock

**Affiliations:** ^1^Burdock Group, Orlando, FL 32801, USA; ^2^The Safety Assessment Division II, Mitsubishi Chemical Safety Institute Ltd., Tokyo 105-0014, Japan; ^3^The Department of Toxicology, Otsuka Pharmaceutical Co. Ltd., Tokushima 771-0192, Japan; ^4^The Saga Nutraceuticals Research Institute, Otsuka Pharmaceutical Co. Ltd., Tokushima 771-0192, Japan

## Abstract

Consumption of the isoflavones daidzein, genistein, glycitein, and their structural analogues is generally considered beneficial to human health. Equol is not found in soy, but is converted from daidzein by human gut bacterial flora. Research indicates that between 30–50% of the population is capable of converting daidzein to equol; therefore, there has been recent development of a new equol-rich functional food that relies on bacterial conversion of daidzein to equol under strictly controlled conditions. Therefore, a new equol-rich soy product (SE5-OH) has been developed, based on the bacterial conversion of daidzein; and its reproductive and developmental toxicity has been evaluated in a two-generation study and a developmental toxicity study with Sprague-Dawley rats at dose levels of 200, 1000, and 2000 mg/kg/day by gavage. SE5-OH contains approximately 0.65% equol, 0.024% daidzein, 0.022% genistein, and 0.30% glycitein. From the reproductive study, the no-observed-adverse-effect-level (NOAEL) for SE5-OH determined for both male and female rats is 1000 mg/kg/day (6.5 mg equol/kg/day). In the developmental toxicity phase of the study, no effects by SE5-OH were found in the embryo-fetus at any of the doses tested. The NOAEL for developmental effects of SE5-OH is 2000 mg/kg/day (13 mg equol/kg/day).

## 1. Introduction

Over the past two decades, scientific literature has indicated that consumption of soy products and, in particular, their isoflavone components have been associated with the reduction of the risk of cancer, coronary vascular disease, osteoporosis, and other pathologies that are associated with menopause and ageing in both sexes [1–3]. One such isoflavone is equol: a microbial metabolite of daidzein, which is structurally related to 17*β*-estradiol [4]. Consumption of daidzein in subjects that were found to be able to metabolize daidzein to equol has been linked with delay in the onset of menopause and menopausal-associated symptoms, markers of cardiovascular disease, and prostate cancer [5, 6]; however, there is conflicting evidence concerning the ability to produce equol and subsequent health benefits [6, 7]. Although there are known dietary sources of equol, the levels of equol in these milk samples are relatively low (in the range of 60–450 ng equol/mL) [8, 9]. Therefore, the consumption of equol-enriched soy-based products may be of health benefit to individuals who do not convert daidzein to equol.

**Figure 1:**
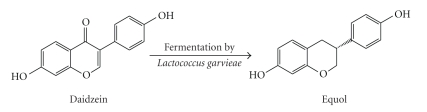
Daidzein metabolism to equol.

In nature, equol is formed exclusively by the intestinal microbiota although chemical synthesis pathways have also been described [10, 11]. The enzymatic process of producing equol from daidzein resides with the gut microbial flora [12, 13], similar to the pathway presented in Figure 1. Although it has been estimated that there are more than 400 species of bacteria in the human gut that may influence the normal structure and function of the intestinal system [14, 15], research indicates that, depending upon the population studied, between 30–50% of the population is host to bacteria which metabolize daidzein into equol [6, 16, 17]. Recent research has indicated that humans that host bacteria, able to metabolize daidzein to equol, convert approximately 30–40% of daidzein into equol, and the types of food consumed may be one of several factors that affect the ability to produce equol [18–20]. Serum levels of equol in equol producers that consume daidzein have been noted at up to 76 ± 137 nmol/L.

The content of equol in the soy-based product (SE5-OH) has been enriched as a result of fermentation of soy germ with the bacterium *L. garvieae* (Otsuka Pharmaceutical Co., Ltd., Chiyoda-ku, Tokyo, Japan). *L. garvieae* is not a normal member of the human gut flora, but is typically found in fish, and within fish aquacultures, and is associated with decreased growth rates, an unmarketable appearance, and mortality, but is not known to produce toxins [21]. *L. garvieae* is a rare pathogen with a low virulence in humans with gastrointestinal disorders that consumed raw fish, also has been found in several different cheeses, and may be an important fermentation microbe in the production of several cheeses, including artisanal Italian and traditional Egyptian Domiati cheeses [22–25]. The controlled process of soy fermentation with *L. garvieae* is capable of converting approximately 50% of the daidzein contained within the soy to equol. SE5-OH is autoclaved to kill the bacteria, and subsequently analyzed to ensure that SE5-OH does not contain any living *L. garvieae*; therefore, the live organism will not be ingested by the consumer.

Previously, we have reported acute and subchronic toxicity studies of SE5-OH in the adult rat model, and determined the NOAEL to be 2000 mg/kg/day following administration for 90 days [26]. The objective of the studies described herein was to investigate the effect of the specific equol-enriched soy-based SE5-OH product on rat development and reproduction over two generations.

## 2. Methods and Materials

### 2.1. Test Articles

The test article, referred to as SE5-OH, was prepared by a controlled fermentation of a daidzein-rich soy germ containing isoflavones with *L. garvieae* (Otsuka Pharmaceutical Co., Ltd., Chiyoda-ku, Tokyo, Japan). SE5-OH contains approximately 0.65% equol, 0.024% daidzein, 0.022% genistein, and 0.30% glycitein. The test article is a light brown powder that includes five specific isoflavones; the amounts in the two lots of test article used in these studies are presented in Table 1. The test article control was hydroxypropylmethylcellulose (HPMC), supplied by Shin-Etsu Chemical Co., Ltd (Chiyoda-ku, Tokyo, Japan).

### 2.2. Animals

Male and/or female Crl:CD(SD) rats, supplied by the Atsugi Breeding Center of Charles River Laboratories (CRL) Japan, Inc., (Kohoku-ku, Yokohama, Japan) were used for both the developmental and reproductive toxicity studies. All animals were clinically monitored at the time of delivery to the testing facility and during the acclimation period. Housing consisted of stainless steel or polycarbonate cages, with a twelve-hour light period (7:00 AM to 7:00 PM). Temperature was maintained at 20.1–23.4°C with a relative humidity of 49.6–69.2% and 6–20 air changes/h. The bedding, which was used only in the reproductive toxicity study, was of the *Beta*-Chip brand supplied by Charles River Laboratories Japan, Inc. The rats were six weeks of age at the start of dosing in the reproductive toxicity study and twelve weeks of age at mating in the developmental toxicity study. Rats were acclimatized for five or six days before use.

### 2.3. Guidelines

The developmental toxicity and reproductive toxicity studies were carried out under the guidelines of *Redbook 2000* and FDA's *Toxicological Principles for the Safety Assessment of Food Ingredients* [27, 28]. Both studies were conducted within the Good Laboratory Practice (GLP) standards for the conduction of nonclinical studies on the safety of drugs (Ministry of Health and Welfare (MHW), Japan, Ordinance Number 21, 26 March 1997, [http://pdg-a258.koyosha.co.jp/jsqa2/en/glp/glp424e.html]). This study was approved by the Committee of Kashima Laboratory for Ethics in Animal Studies in compliance with “Guidelines for Animals Studies (Kashima Laboratory, Mitsubishi Chemical Safety Institute Ltd., Kamisu-shi, Ibaraki, Japan).”

### 2.4. Experimental Design

#### 2.4.1. Developmental Toxicity Study

**(1) Exposure and Observations**

On gestational days (GDs) 6–19, three groups of 22 pregnant females were administered doses of SE5-OH in 1% aqueous HPMC by gavage at levels of 200, 1000, and 2000 mg/kg/day, respectively; the control group (22 pregnant females) was administered 1% HPMC vehicle only. Animals were observed twice daily for clinical signs during the dosing period, and once daily following the dosing regimen. Body weights and gross weights of the diets were measured on GD 0, 6, 8, 10, 12, 14, 16, 18, and 20. Body weight gain was calculated on the basis of body weights measured on GD 6.

**(2) Necropsy**

On GD 20, dams were euthanized, the gravid uterus was collected from each dam and weighed to allow for calculation of corrected body weight and corrected body weight gain. Corrected body weight was determined by subtracting the gravid uterine weight from the body weight on GD 20, while the corrected body weight gain was calculated by subtracting the body weight on GD 6 from the corrected body weight. At necropsy of the dams, thoracic and abdominal organs and tissues were examined macroscopically. Any organs or tissues showing abnormalities were collected and preserved. If such collections were conducted, the same tissues were collected from three control dams and also preserved. Ovaries and uterus were removed and examined for the number of corpora lutea, implantations, live fetuses, and dead embryos. Early embryonic deaths were considered to be represented by the number of implantation sites plus placental remnants; late embryonic deaths were represented by macerated embryos plus dead fetuses.

Live fetuses were weighed, euthanized, sexed, and examined (external and oral cavity examinations). Approximately half of the live fetuses in each litter from the control and the 2000 mg/kg dose dams were subjected to visceral examination, which included the head, neck, thorax, and abdomen. The remaining fetuses were examined for skeletal anomalies and degrees of ossification in cervical vertebral bodies, sternebrae, metacarpi, metatarsi, and sacral/caudal vertebral bodies.

#### 2.4.2. Reproductive Toxicity Study

Groups of 52 rats (26/sex/group) served as the F_0_ generation and were administered the same SE5-OH test article at the same doses via gavage as in the developmental toxicity study (i.e., 0, 200, 1000, and 2000 mg/kg/day). Dosing commenced at six weeks of age and lasted for ten weeks before mating and then throughout the mating period. The mating procedure involved the placement of one male and one female in the same cage for a maximum of two weeks, with reproductive capacity judged by copulation index, fertility index, precoital period, gestation period, and gestation index. F_0_ females that became pregnant continued on this exposure regimen through gestation and lactation until weaning of the F_1_ animals. The F_1_ generation males and females were also administered the same dosing regimen of SE5-OH, beginning on postnatal day (PND) 21, for ten to eleven weeks before mating and throughout the mating period, with pregnant females continuing on the dosing schedules through gestation and lactation until weaning of the F_2_ generation. Throughout this entire process, animals were observed twice daily for clinical signs; body weights and food consumption were measured weekly until the start of gestation in both F_0_ and F_1_ generations. Estrous cycle was examined for three weeks before the start of mating in both F_0_ and F_1_ females. During gestation, females in both generations were weighed on GD 0, 3, 7, 10, 14, 17, and 20 and then on lactation day (LD) 0, 4, 7, 10, 14, 17, and 21 during lactation. Food consumption was also measured during GD 0, 7, 14, and 20 and on LD 0, 4, 7, 14, and 21. On the day after the animals reached the end of their respective exposure periods, they were euthanized for gross necropsy. The following tissues were weighed and preserved: adrenals, brain, kidney, liver, female reproductive tissues (uterus, ovaries), male reproductive tissues (testes, epididymis, prostate, and seminal vesicles including coagulating glands), pituitary, spleen, thymus (neonatal only), and thyroid gland. Histological examinations were conducted on tissues from control and high- (2000 mg/kg) dose groups only and included adrenals (F_0_ females were examined in all dose groups), male and female reproductive organs, pituitary, and liver tissues from F_0_ females. Sperm was also collected from the cauda epididymis of all F_0_ and F_1_ males and evaluated for motility, and the sperm from the control and 2000 mg/kg dose group were also evaluated for spermatid cells (testis), sperm number, and morphology.

Clinical parameters for offspring included examination for external anomalies, sex ratios, live birth index (i.e., number of live pups/number of total pups born), as well as viability index on PND 4 and weaning index on PND 21; pup weights were measured at birth as well as PND 4, 7, 14, and 21. On PND 4, litter sizes were adjusted randomly to eight pups per dam. Pups were also examined for physical and functional developmental parameters, which included incisor eruption, eye opening, reflexes for righting, auditory startle, pain, corneal, and pupillary reactions. Sexual maturation was determined in F_1_ rats by recording the age and weight on the day of vaginal opening after PND 27 was reached or when balanopreputial separation from PND 35 was recorded. At gross necropsy of F_1_ and F_2_ weanlings, brain, spleen, and thymus were collected from selected animals for preservation (one male and one female selected from each litter in numerical order of the offspring number in each litter, which was randomly assigned). Ovaries, vagina, and uterus were collected from females, while testes, epididymis, seminal vesicles, and prostate were collected from males. Organ weights were determined from one animal of each sex per litter for brain, thymus, spleen, and uterus.

### 2.5. Statistical Analyses

Statistical analysis for the reproductive and developmental toxicity study was conducted using several different tests, using the toxicological data processing system (MiTOX, Mitsui Zosen Systems Research, Inc., Japan). The data of offspring obtained before weaning were analyzed on the basis of litter mean values except for the sex ratio. The normality of the data was assumed, unless otherwise indicated. Homogeneity of variance was tested by Bartlett's test; when the data were homogeneous, a one-way analysis of variance (ANOVA) was performed for statistical comparison. Heterogeneous data were tested by the Kruskal-Wallis test. When a significant intergroup difference was found, Dunnett's or the Dunnett-type multiple comparison test was used. For some of the data (e.g., days until copulation, number of estrus stages without copulation, gestation length, sperm motility, birth index, viability index on PND 0, 4, and 21, incidence of offspring with external anomalies, postnatal physical development, reflex and sensory function, as well as sexual maturation (vaginal opening and cleavage of balanopreputial gland)), the Kruskal-Wallis test was applied first, and when a significant intergroup difference was found, the Dunnett-type multiple comparison test was used. The multiple comparison test was utilized to evaluate statistical significance of changes in body weight, body weight gain, food consumption, organ weights, number of implantations, number of offspring, number of live offspring, and estrous cycle lengths. However, for the spermatid count and sperm count, the homogeneity of variance was tested by the F test, and when the variance was homogeneous, Student's *t*-test was performed for the statistical comparison. When heterogeneous, the Aspin-Welch *t*-test was used. Moreover, Wilcoxon's rank sum test was used for comparison of the incidence of abnormal sperm (morphologically abnormal sperm and tailless sperm), visceral examination, and skeletal examinations. The categorical data (e.g., copulation index, fertility index, gestation index, sex ratio (male/female), incidence of females with irregular estrous cycles, and dams with external abnormal offspring) were analyzed by Fisher's exact probability test. All statistical tests used two-tailed significance level of 5%.

## 3. Results

### 3.1. Developmental Toxicity Study

#### 3.1.1. Effects on Dams

**Figure 2:**
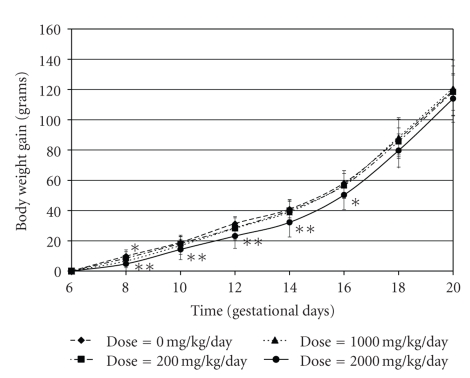
Changes in body weight gain (grams) observed in dams exposed to SE5-OH during gestational days 0–20 during the developmental toxicity study in rats; significant when compared to control: *P < .05; **P < .01

With the exception of a transient lack of weight gain (see Figure 2), no abnormal clinical signs were observed in any of the dams. This included observations for death, moribundity, abortion, or premature delivery (data not shown). In dams exposed to 2000 mg/kg, significant decreases in body weight gain were noted on GD 8–16 but these values had returned to control levels by GD 20. The same transient lack of weight gain was noted for dams in the 1000 mg/kg exposure group, but only on GD 8. No treatment-related effects were seen for food consumption, gravid uterine weights, corrected body weight, and corrected body weight gain; and there were no abnormalities seen at necropsy (data not shown).

#### 3.1.2. Effects on Embryo-Fetal Development

In all treated groups, there were no significant differences in number of corpora lutea, implantations, dead embryos, postimplantation losses, live fetuses, fetal deaths, fetal weight, or sex ratio. Several isolated embryo-fetal effects were noted during the placental observations, and external, visceral, and skeletal examinations. These changes are summarized in Table 2.

### 3.2. Reproductive Toxicity Study

#### 3.2.1. Parental Animals

Transient decrease of weight gain and reduced food intake in the F_0_ and F_1_ females of 2000 mg/kg/day, and histological change of adrenal cortex (a diffuse increase in lipid droplets in the zona glomerulosa) in the F_0_ females of 2000 mg/kg/day were noted (see Table 3). Food intake was reduced in the F_0_ females in the 1000 mg/kg/day group. In addition, increased liver weight in the F_0_ female of 1000 and 2000 mg/kg/day dose groups, increased adrenal weight in the F_0_ males at the 2000 mg/kg/day dose group, as well as increased thyroid weight in the F_1_ males (see Table 4) was noted. No adverse clinical signs (including death, moribundity, abortion, or premature delivery), necropsy findings, or organ weights were observed in any of the F_0_ and F_1_ parental animals.

There were no adverse reproductive effects observed in any treatment group of F_0_ and F_1_ animals in terms of estrous cycle, copulation index, fertility index, or precoital period (see Tables 5 and 6). No adverse effects were seen in gestation period, gestation index, or parturition. No effect on implantation number was found in the F_0_ generation although the number of implantations was slightly, but significantly, reduced in the F_1_ dams at 2000 mg/kg/day, there were no effects on implantation in the 200 and 1000 mg/kg/da F_1_ generation (see Tables 7 and 8). The decrease in implantation number in the high dose did not significantly affect the birth index or the number of offspring born alive. SE5-OH administration to male rats did not affect any of the sperm parameters evaluated, which included sperm motility, percentage and type of abnormal sperm, tailless sperm, sperm count, or the number of homogenization-resistant sperm (see Table 9).

#### 3.2.2. Offspring Development

Reproductive performance for the F_0_ generation was initiated with 26 mating pairs. While not statistically significant, 2, 2, and 1 pairs in the 200, 1000, and 2000 mg/kg/day dose groups, respectively, did not copulate. In the copulating pairs, nonpregnant females were observed in 1, 2, and 3 cases of the control, 1000, and 2000 mg/kg/day groups, respectively; again, this did not result in a significant difference in the fertility index. The F_0_ breeding resulted in 25, 24, 22, and 22 dams in the control, 200, 1000, and 2000 mg/kg/day dose groups, respectively. The number of litters delivered resulted in 24, 23, 22, and 22 litters/dose in the control, 200, 1000, and 2000 mg/kg/day dose groups, respectively (see Table 10). One complete litter in the 1000 mg/kg/day dose group was lost on PND 2.

For F_1_ rat pups, one male and one female were randomly selected from each litter on PND 21 and were used as breeding animals in the F_1_ generation (siblings not paired). One male pup in the control group died on PND 21, and one female 1000 mg/kg/day pup died on PND 22. In addition, two F_1_ females in the control group and one F_1_ male in the 1000 mg/kg group that had no mating partner were not subjected to mating. This resulted in 22, 23, 20, and 22 breeding F_1_ pairs in the control, 200, 1000, and 2000 mg/kg/day dose groups, respectively. All mating pairs copulated except for one pair in each of the control and 200 mg/kg/day groups. Three, 3, 1, and 1, nonpregnant females occurred in control, 200, 1000, and 2000 mg/kg/day dose groups, respectively. One female died with vaginal hemorrhage on GD 23 in the 200 mg/kg/day dose group, which was not considered to be test article-related. One male of the control group could not fertilize the mating female, and one male of the 2000 mg/kg/day dose group exhibited unilateral small and soft testis and small epididymis, and the mated female of this male had only one implantation with no delivery. These effects were not significant and not considered to be related to test article administration, as they were not dose-related responses. Of the dose groups, 18, 18, 19, and 21 F_1_ dams gave birth to live F_2_ offspring (see Table 11). There were no changes attributable to the test article in parturition or nursing. Total litter loss was observed for one dam each in the 200 and 2000 mg/kg/day dose groups on PND 4 and PND 1, respectively; however, this was judged to be incidental as this was not a dose-dependent effect.

In the F_2_ generation, the number of female offspring was reduced at the 2000 mg/kg/day dose on PND 4 (*P* < .05), but neither the number of live offspring on PND 21 nor the viability index was affected. This reduction was not caused by prenatal death or a difference in the birth index, but was only a reflection of a nonsignificant reduction in the number of implantations. The total litter loss on PND 1 was judged to be incidental, as there was no dose dependency in the incidence and no abnormalities in the maternal behavior in the dams. There were no adverse effects observed in any treatment group of F_1_ and F_2_ offspring: clinical signs, body weight, external anomalies, sex ratios, viability index, physical development, reflex function, organ weight, necropsy findings, and sexual maturation (data not shown), or viability index (see Table 11).

## 4. Discussion

Soy is the most widely used food plant in the world and has been cultivated for over 4500 years, resulting in human soy isoflavone consumption for several millennia [29]. Recently published data by the USDA [30] indicate that daidzein, genistein, and glycitein are constituents in a wide variety of legumes, prepared foods, spices, teas, and of course, soy-based foods, including infant formula, tofu, tempeh, cheese, beverages, noodles, sauces, chips, and meat substitutes. In unfermented food products, the glycosylated forms (i.e., daidzin, genistin, glycitin, and their derivatives) are more abundant than the aglycone forms. The widespread historical exposure to soy isoflavones in the diet, without adverse human health effects, and the overall body of available scientific evidence, is a compelling evidence of their safety. While average Japanese isoflavone intake has been estimated at 50 mg/day, western consumption is approximated at 5 mg/day [31, 32].

There has been a concern that ingestion of soy isoflavones may lead to adverse effects on testes and ovaries [33–36]. Unlike diethylstilbestrol (DES), equol at 1000 *μ*g/animal/day injected subcutaneously for five days failed to demonstrate estrogenic, or antiestrogenic activity in the uterine wall of neonatal Sprague-Dawley (SD) rats [37]. Other research found that equol administration directly to the uterine horn in ovariectomized rats did not increase uterine weight, and although equol did weakly bind to the estrogen receptor with a high dissociation rate, it did not demonstrate antiestrogenic activity [38]. In concert with findings reported by Thompson et al. [38], Wood et al. [39] found that high doses of genistein and daidzein or equol had minimal uterotrophic or mammotrophic effects in an ovariectomized monkey (*Macaca fascicularis*) model. In the normal postmenopausal mammary gland of cynomolgus macaques, equol at an equivalent human dose of 105 mg/day for greater than six months lacked estrogenic effects, and did not promote lesions in the mammary tissue [40]. Rachon et al. [41–43] found that equol in the diet at 400 mg per kg of food (delivering approx. 26.16 mg equol/kg body weight/day to the rat) for three months to ovariectomized rats significantly increased serum prolactin and lutenizing hormone levels (*P* < .05), but had no effect on pituitary estrogen receptor (ER)-*α* or -*β* gene expression. However, equol administration did increase an isoform of the estrogen receptor (Terp-1) in the pituitary of these ovariectomized rats, and increased the number of terminal ducts and type II lobules, compared to control rats. Equol dosed at 3.68 mg/kg/day in this same study had no effect on rat mammary tissue. In this same rat model, equol (26.16 mg/kg body weight/day) significantly increased uterine weights and increased the epithelial height and thickness of the uterine stroma and myometrium, and significantly increased levels of uterine insulin-like growth factor 1, progesterone receptor, and complement protein 3 mRNA, while 3.68 mg equol/kg bodyweight/day had no effect [44]. Subcutaneous administration of up to 1000 *μ*g equol in rat pups during PND 1–5 or PND 10–14 lowered uterine weight at later ages, but did not affect ER levels [45]. Equol administration (approx. 27.48 mg/kg body weight/day) in ovariectomized rats for six weeks increased uterine weight, decreased weight gain, and attenuated trabecular bone loss (but not proximal tibia bone loss), while increasing its density, indicating mild bone sparing effects [46]. These studies indicate that there is an uncertain degree of difference in species- and organ-specific sensitivity to equol administration.

SE5-OH is a unique fermented soy germ food product that is intended to deliver equol to individuals who cannot produce it endogenously. The studies currently described were conducted to determine any potential developmental and reproductive effects of SE5-OH. In the developmental study, isolated embryo-fetal effects were noted during necropsy, which included the fusion of placentae in one dam in the 200 mg/kg/day dose group. As no other placental abnormalities were identified in this study, this effect was determined not to be test article-related. One control group fetus and one high-dose fetus were found to have a ventricular septal defect, and visceral variants that included a thymic neck remnant, supernumerary coronary ostium, left umbilical artery, and renal pelvis dilations which occurred with equal incidence in these dose groups and were determined not to be test article-related. Dumbell ossification of the thoracic centrum was significantly lower in the high-dose group, but the overall incidence of the observed skeletal variants (as indicated in Table 4) was determined not to be test article-related because they are similarly seen in untreated Crl:CD(SD) rats.

An increase in relative (but not absolute) liver weight was noted in the F_0_ 1000 and 2000 mg/kg/day dams, and in the 2000 mg/kg/day F_1_ dams, but was not seen in the male rats. The increase at the 1000 mg/kg/day dose did not exceed the limits of 5% variability, which is generally considered within the accepted level of variability to the control rats. The relative liver weight at the 2000 mg/kg/day dose had greater than 5% variability from controls. There was a significant increase in the absolute and relative thyroid weight in the 1000 mg/kg/day dose group, but this was not considered related to SE5-OH treatment, as this effect was not noted in any other dose levels. The highest dose of SE5-OH (2000 mg/kg/day) had some indications of toxicity at the gestational and conception stages in the rat model.

The adrenal cortex of F_0_ females at the 2000 mg/kg/day SE5-OH dose had an increased number of diffuse lipid droplets (4/10 in the high-dose group versus 0/10 in the controls) noted in the glomerular zone of the adrenal glands. Lipid droplets are the intracellular stores of cholesterol esters, precursors of hormones formed in the rat adrenal glands [47]. It has been documented that cell enlargement and widening of the zona glomerulosa of the adrenal cortex (associated with an increase in lipid droplet volume) may be induced via androgen receptor binding, and that isoflavones (including equol as an antiandrogen [48]) have been reported to alter androgen receptor expression and adrenocortical function [49, 50]. However, the exact mechanism is not known, as isoflavones have been shown to increase or decrease androgen production, depending on the model used [51–54]. Wood et al. [53] noted that consumption of a soy protein providing 129 mg/day isoflavones (which provided approx. 31 mg daidzein per day) by female cynomolgus monkeys for 36 months did not alter serum androgen levels, but decreased adrenal weight (*P* < .0006). However, previous postmenopausal oral contraceptive administration abolished this effect (*P* < .01). Diffuse lipid droplets in the adrenal cortex have been noted in the rat model as a consequence of advancing age [47], and coupled with pregnancy, they may have been a factor in the results of this study. The maximal plasma concentration was 390 ± 90 and 414 ± 32 ng/mL, respectively, for the S- and R-enantiomers.

Although a significant reduction in the number of implantations in this study is thought to have occurred due to lowered caloric intake in the high-dose pregnant dams, it still remains that there was an overall adverse effect, albeit an indirect one (i.e., an effect of reduced caloric intake on decreased body weights) and not a product of the intrinsic toxicity of SE5-OH on the reproductive ability of the rats in this study. Even though a transient decrease in body weight gain was noted in F_0_ and F_1_ dams of the 2000 mg/kg/day dose group, no changes were observed in fetal viability, intrauterine growth, and fetal morphology (external, skeletal, and visceral) that were attributed to SE5-OH treatment. Isolated embryo-fetal alterations were noted, but were not consistent with SE5-OH treatment or significantly different from controls. It is known that food restriction affects the adult female rat reproductive capacity and may decrease the number of corpus luteum graviditatis [55, 56]. Equol administration to ovariectomized rats at approximately 27.48 mg/kg/day for six weeks [44] resulted in decreased weight gain, intra-abdominal fat, plasma leptin, and total cholesterol levels. Soy isoflavone administration to male, female, and ovariectomized rats reduced food intake and weekly body weight gain, while increasing serum isoflavones, including equol [57]. A decrease in implantations was noted at the 2000 mg/kg/day dose group; however, since no adverse effects were noted on organ weights or histopathological examination of the reproductive organs, mating ability, fertility, pregnancy, parturition, or nursing behavior in F_0_ or F_1_ animals, this slight decrease in implantation number was not a substantive or meaningful effect. Further, the absence of an effect on the fecundity of the high-dose animals in the reproduction study indicates this decrease in implantation was an anomalous finding, and is supported by the lack of reproductive toxicity by daidzein in rats reported by Lamartiniere et al. [34]. In a previous work [26], no adverse observations were found in adult rats at up to 2000 mg/kg/day SE5-OH in a subchronic 90-day study. In the present study, no adverse toxicological observations were made at either the reproductive or developmental stages in the either sex of the rat model at the 1000 mg/kg/day dose. From the combined studies herein, we can conclude that in the developmental toxicity phase of the study, a no-observed-adverse-effect-level (NOAEL) for developmental effects of SE5-OH is 2000 mg/kg/day, based on the lack of significant embryo-to-fetal stage effect. From the reproductive study, the NOAEL for SE5-OH determined for both male and female rats is 1000 mg/kg/day (6.5 mg equol/kg/day), based on the reduction of body weight, implantations, and live births in the F_1_ and F_2_ animals at the 2000 mg/kg/day dose level.

## Figures and Tables

**Table 1:** Isoflavone constituents of SE5-OH.

Isoflavone ingredient*	Reproductive toxicity study (Lot 060106P)** (mg/g)	Reproductive toxicity study and developmental toxicity study (Lot 051206P)** (mg/g)*
Daidzein	0.323 ± 0.0015	0.275 ± 0.0025
Genistein	0.293 ± 0.0017	0.287 ± 0.0032
Glycitein	1.65 ± 0.015	2.04 ± 0.010
Dihydrodaidzein	0.362 ± 0.0021	0.337 ± 0.0036
Equol	5.16 ± 0.070	5.24 ± 0.025

*The amount of each isoflavone ingredient is the average of three separate measurements, mean ± standard deviation.

**Both lots were used in the reproductive study, while only Lot 051206P was used in the developmental study.

**Table 2:** Isolated embryo-fetal effects noted during necropsy observations/examinations in the developmental toxicity study in rats.

Effect	Occurrence
Fusion of placentae	Occurred in one dam in the 200 mg/kg dose group. No other placental abnormalities identified in any group.
Rudimentary tail	Occurred in one fetus of the 1000 mg/kg dose group. No other external fetal abnormalities identified in any group.
Visceral anomaly: ventricular septal defect	Occurred in one control group fetus and one 2000 mg/kg group fetus. No other anomalies identified in any group.
Visceral variants: thymic neck remnant, supernumerary coronary ostium, left umbilical artery, and renal pelvis dilation	Occurred with equal incidence in the control and 2000 mg/kg groups.
Skeletal anomalies/malformations: wavy and nodulated ribs	Occurred in one 2000 mg/kg group fetus. No other anomalies identified in any group.
Skeletal variants: foramen hypoglossi double, closure of transverse foramen of one or more cervical vertebral arches, transverse foramen opening in 7th cervical arch, splitting of ossification centers in thoracic vertebral bodies, hemicentric thoracic centrum, dumbbell ossification of thoracic centrum, lumbarization, cervical ribs, short supernumerary 14th rib, reduced 13th ribs, splitting of sternebrae	Incidence of dumbbell ossification of thoracic centrum was significantly low in the 2000 mg/kg dosing group but was judged incidental with no toxicological significance.

**Table 3:** Body weight and food consumption in male and female Sprague-Dawley rats during the two-generation reproductive toxicity study.

Generation				F_0_				F_1_			
Dose (mg/kg/day)	Gender		Period (days)	0	200	1000	2000	0	200	1000	2000
Body weight (g)	Male	Premating	0	195.9 ± 6.6	196.5 ± 5.7	196.2 ± 6.3	195.5 ± 6.2	59.6 ± 4.9*#*	59.7 ± 5.0	59.4 ± 5.7	57.1 ± 5.1
			70	540.0 ± 47.2	560.1 ± 56.6	534.3 ± 46.4	539.3 ± 41.2	451.8 ± 35.1	461.±32.1	442.8 ± 37.8	445.5 ± 34.9
				(*n* = 26)	(*n* = 26)	(*n* = 26)	(*n* = 26)	(*n* = 23)	(*n* = 23)	(*n* = 21)	(*n* = 22)
	Female	Premating	0	152.2 ± 8.5	152.5 ± 6.9	151.0 ± 6.9	151.2 ± 6.8	55.4 ± 6.0	57.0 ± 5.8	57.6 ± 4.8	54.8 ± 5.5
			70	303.9 ± 35.6	298.3 ± 21.5	289.2 ± 22.4	283.0 ± 19.1*	266.6 ± 22.9	270.0 ± 26.8	263.2 ± 21.0	252.9 ± 17.1
				(*n* = 26)	(*n* = 26)	(*n* = 26)	(*n* = 26)	(*n* = 24)	(*n* = 23)	(*n* = 20)	(*n* = 22)
		Gestation	7	337.9 ± 28.8	333.9 ± 21.3	323.3 ± 22.7	318.5 ± 21*	358.9 ± 32.3	367.1 ± 34.9	352.6 ± 29.6	334.7 ± 20.7*
			14	371.0 ± 29.5	364.0 ± 23.3	355.4 ± 23.8	349.3 ± 23.4*	395.4 ± 34.4	400.9 ± 35.5	383.4 ± 29.6	367.6 ± 22.9*
			20	443.0 ± 36.4	440.2 ± 25.4	434.7 ± 26.7	428.9 ± 23.6	474.9 ± 40.5	473.3 ± 39.1	453.9 ± 38.9	437.3 ± 34.9**
				(*n* = 25)	(*n* = 24)	(*n* = 22)	(*n* = 22)	(*n* = 18)	(*n* = 19)	(*n* = 19)	(*n* = 21)
		Lactation	21	355.0 ± 24.2	345.8 ± 20.4	345.3 ± 21.7	343.0 ± 19.5	365.3 ± 25.6	370.8 ± 28.0	368.0 ± 22.8	355.8 ± 14.8
				(*n* = 24)	(*n* = 23)	(*n* = 21)	(*n* = 22)	(*n* = 18)	(*n* = 17)	(*n* = 18)	(*n* = 19)

Body weight gain (g)	Male	Premating	0–70	344.1 ± 45.1	363.6 ± 54.4	338.1 ± 43.7	343.8 ± 40.0	392.3 ± 34.1	401.8 ± 30.2	383.4 ± 34.1	388.4 ± 32.1
	Female	Premating	0–70	151.7 ± 31.1	145.9 ± 20.4	138.2 ± 19.3	131.9 ± 16.5*	211.2 ± 20.8	213.0 ± 25.0	205.4 ± 19.3	198.1 ± 16.9
		Gestation	0–7	33.2 ± 7.1	31.3 ± 5.5	29.6 ± 8.9	30.7 ± 7.9	36.8 ± 7.7	34.6 ± 8.7	32.1 ± 9.3	33.3 ± 7.9
			0–14	66.3 ± 12.7	61.4 ± 8.8	61.7 ± 8.0	61.5 ± 12.4	73.2 ± 8.4	68.4 ± 12.1	62.8 ± 10.7*	66.2 ± 11.8
			0–20	138.3 ± 22.3	137.6 ± 16.4	141.0 ± 11.0	141.0 ± 18.5	152.8 ± 13.0	140.8 ± 24.5	133.3 ± 20.8**	136.0 ± 26.5
		Lactation	0–21	1.6 ± 20.3	−2.4 ± 20.2	6.8 ± 20.8	3.4 ± 13.9	−20.3 ± 20.5	−17.4 ± 25.4	−7.7 ± 23.4	−5.5 ± 23.0

Food consumption (g)	Male	Premating	70	26.2 ± 1.9	26.9 ± 1.8	25.4 ± 1.6	25.1 ± 1.8	30.5 ± 2.0	31.3 ± 1.8	29.0 ± 1.8	28.9 ± 1.9
	Female	Premating	70	19.0 ± 1.7	18.5 ± 1.6	17.6 ± 1.2*	16.3 ± 0.9**	20.3 ± 2.1	20.6 ± 1.5	19.2 ± 1.3	17.5 ± 1.1**
		Gestation	7	22.6 ± 1.7	22.1 ± 1.9	20.7 ± 2.1**	20.4 ± 2.4**	24.4 ± 1.9	24.3 ± 2.9	22.6 ± 3.1	21.4 ± 2.1**
			14	24.8 ± 2.4	24.2 ± 2.2	23.1 ± 2.0*	22.8 ± 2.3**	26.3 ± 2.2	26.3 ± 2.8	24.7 ± 2.6	23.7 ± 2.6**
			20	26.0 ± 2.4	25.7 ± 2.6	26.0 ± 2.2	25.1 ± 2.1	27.7 ± 2.1	26.3 ± 2.5	25.8 ± 2.1	26.4 ± 2.6
		Lactation	21	68.4 ± 8.5	68.8 ± 3.7	69.4 ± 4.0	67.4 ± 5.4	67.2 ± 6.6	67.9 ± 5.3	64.1 ± 6.4	65.7 ± 9.2

Mean ± standard deviation; significantly different from control: *P < .05, **P < .01; *#* the start of dosing for the F_0_ groups was at six weeks of age. The start of dosing for the F_1_ group was on day 21 postparturition, to maintain SE5-OH ingestion via lactation; *n*: number of male and female rats per dose group.

**Table 4:** Organ weight changes in male and female breeding Sprague-Dawley rats in the F_0_ and F_1_ generations of the two-generation rat study.

Generation	F_0_				F_1_			
Dose (mg/kg/day) (*n*)	0 (*n* = 26)	200 (*n* = 26)	1000 (*n* = 26)	2000 (*n* = 26)	0 (*n* = 22)	200 (*n* = 22)	1000 (*n* = 20)	2000 (*n* = 26)
Male								

Thyroids, absolute organ weight (mg)^A^	30.01±5.45^C^	31.65 ± 5.94	29.94 ± 3.88	30.32 ± 3.95	30.19 ± 4.48	31.89 ± 4.91	35.15±5.27**	32.09 ± 4.18
Thyroids, relative organ weight (mg)^B^	5.23 ± 0.68	5.33 ± 0.86	5.33 ± 0.73	5.34 ± 0.78	4.55 ± 0.61	4.76 ± 0.73	5.48±0.90**	4.94 ± 0.58
Adrenals, absolute organ weight (mg)	61.81 ± 7.05	63.95 ± 10.16	59.48 ± 7.40	68.90±9.01**	62.18 ± 7.30	63.96 ± 5.83	57.61 ± 7.52	62.28 ± 9.25
Adrenals, relative organ weight (mg)	10.88 ± 1.31	10.82 ± 1.68	10.57 ± 1.28	12.10±1.58**	9.41 ± 1.14	9.56 ± 1.07	8.95 ± 1.08	9.62 ± 1.57
Seminal Vesicles, absolute organ weight (g)	3.11 ± 0.33	2.90 ± 0.37	3.12 ± 0.37	3.05 ± 0.34	3.40 ± 0.29	3.26 ± 0.44	3.23 ± 0.39	3.13 ± 0.37
Seminal vesicles, relative organ weight (g)	0.55 ± 0.07	0.49±0.09*	0.55 ± 0.08	0.54 ± 0.06	0.52 ± 0.08	0.49 ± 0.01	0.51 ± 0.08	0.48 ± 0.06

Generation	F_0_				F_1_			
Dose (mg/kg/day) (*n*)	0 (*n* = 26)	200 (*n* = 26)	1000 (*n* = 26)	2000 (*n* = 26)	0 (*n* = 18)	200 (*n* = 17)	1000 (*n* = 18)	2000 (*n* = 19)

Female								

Liver, absolute organ weight (g)	13.70 ± 1.30	13.99 ± 1.08	14.39 ± 1.62	14.48 ± 1.11	15.01 ± 1.28	15.45 ± 1.86	16.02 ± 1.46	15.71 ± 1.35
Liver, relative organ weight (g)	3.86 ± 0.32	4.05 ± 0.31	4.17±0.35**	4.23±0.29**	4.12 ± 0.34	4.17 ± 0.41	4.36 ± 0.33	4.42±0.33*

Significantly different from control: *P < .05, **P < .01; ^A^absolute organ weight is the weight of the organ; ^B^relative organ weight is the weight of the organ relative to the body weight at necropsy (body weight ratio); ^C^mean ± standard deviation; *n*: number of male and female rats per dose group.

**Table 5:** Effect of SE5-OH administration on the reproductive performance of the F_0_ generation rat dams.

Dose (mg/kg/day)	Mean estrus cycle	Incidence of irregular estrus cycle	Mating period		Copulation index (%)^A^	Fertility index (%)^B^
			Number of estrus	Day of conceiving		
0	4.26 ± 0.43*#*		0.0 ± 0.0	2.5 ± 1.8	100.0	96.2
	(*n* = 25)	1/26	(*n* = 26)	(*n* = 26)	(26/26)	(25/26)
200	4.18 ± 0.32		0.0 ± 0.0	2.8 ± 1.6	92.3	100.0
	(*n* = 24)	2/26	(*n* = 26)	(*n* = 24)	(24/26)	(24/24)
1000	4.21 ± 0.40		0.0 ± 0.0	2.9 ± 2.7	92.3	91.7
	(*n* = 26)	0/26	(*n* = 26)	(*n* = 24)	(24/26)	(22/24)
2000	4.15 ± 0.38		0.0 ± 0.0	3.3 ± 2.5	96.2	88.0
	(*n* = 24)	2/26	(*n* = 26)	(*n* = 25)	(25/26)	(22/25)

*#*Mean ± standard deviation; ^A^number of copulated females/number of pairs; ^B^number of pregnant females/number of copulated females; *n*: number of mating pairs.

**Table 6:** Effect of SE5-OH administration on the F_1_ generation rat dam reproductive performance.

Dose (mg/kg/day)	Mean estrus cycle	Incidence of irregular estrus cycle	Mating period		Copulation index (%)^A^	Fertility index (%)^B^
			Number of estrus	Day of conceiving		
0	4.36 ± 0.46*#*		0.1 ± 0.6	3.1 ± 2.8	95.5	85.7
	(*n* = 24)	0/24	(*n* = 22)	(*n* = 21)	(21/22)	(18/21)
200	4.31 ± 0.45		0.2 ± 0.7	2.8 ± 1.8	95.7	86.4
	(*n* = 21)	2/23	(*n* = 23)	(*n* = 22)	(22/23)	(19/22)
1000	4.39 ± 0.41		0.1 ± 0.2	2.3 ± 1.3	100.0	95.0
	(*n* = 20)	0/20	(*n* = 20)	(*n* = 20)	(20/20)	(19/20)
2000	4.30 ± 0.43		0.0 ± 0.0	2.5 ± 1.5	100.0	95.5
	(*n* = 21)	1/22	(*n* = 22)	(*n* = 22)	(22/22)	(21/22)

*#*Mean ± standard deviation; ^A^number of copulated females/number of pairs; ^B^number of pregnant females/number of copulated females; *n*: number of mating pairs.

**Table 7:** Effects of SE5-OH administration on F_0_ generation rat dam gestation and delivery parameters.

Dose (mg/kg/day)		Gestation length (days)	Number of implantation sites	Number of offspring born alive	Birth index^A^ (%)	Gestation index^B^ (%)
0		22.3 ± 0.5	15.0 ± 3.9	13.8 ± 4.1	87.12 ± 21.64	96
	*n*	24	25	25	25
200		22.2 ± 0.4	15.1 ± 3.6	13.8 ± 3.4	89.03 ± 20.48	95.8
	*n*	23	24	24	24
1000		22.3 ± 0.6	15.3 ± 1.7	13.6 ± 3.3	89.27 ± 19.68	100.0
	*n*	22	22	22	22
2000		22.0 ± 0.3	14.7 ± 2.1	13.8 ± 2.3	93.63 ± 9.28	100.0
	*n*	22	22	22	22

Significantly different from control: *P < .05, **P < .01; ^A^birth index = (number of offspring born alive divided by the number of implantations); ^B^gestation index = (number of females with live offspring divided by the number of pregnant females); *n*: number of dams per group.

**Table 8:** Effects of SE5-OH administration on F_1_ generation rat dam gestation and delivery parameters.

Dose (mg/kg/day)	Gestation length (days)	Number of implantation sites	Number of offspring born alive	Birth index^A^ (%)	Gestation index^B^ (%)
0	22.2 ± 0.4	15.8 ± 1.2	14.9 ± 1.9	93.8 ± 7.4	100
	*n* = 18	*n* = 18	*n* = 18	*n* = 18
200	22.2 ± 0.4	15.8 ± 2.5	14.0 ± 2.3	88.9 ± 9.2	100
	*n* = 18	*n* = 18	*n* = 18	*n* = 18
1000	22.2 ± 0.4	13.8 ± 4.1	12.7 ± 3.9	88.1 ± 22.8	94.7
	*n* = 18	*n* = 19	*n* = 19	*n* = 19
2000	22.3 ± 0.6	12.6±4.7*	11.3 ± 5.1	84.5 ± 24.8	95.2
	*n* = 20	*n* = 21	*n* = 21	*n* = 21

^A^Birth index = (number of offspring born alive divided by the number of implantations); ^B^gestation index = (number of females with live offspring divided by the number of pregnant females); *n*: number of dams per group.

**Table 9:** Sperm parameters in control and high-dose male rats used as breeding males in the F_0_ and F_1_ two-generation reproduction study.

Dose (mg/kg/day)	Sperm motility (%)	HRS (×10^6^/g)	Sperm count (cauda epididymal) (×10^6^/g)	Abnormal sperm (%)	Types of abnormal sperm (%) no.					Tailless sperm (%)
					A	B	C	D	E	
F_0_ generation										

0	91.0 ± 4.5	83.73 ± 12.52	534.73 ± 72.79	0.81 ± 1.26	0.25 ± 0.62	0.04 ± 0.14	0.00 ± 0.00	0.42 ± 0.76	0.10 ± 0.40	1.96 ± 2.03
*n*	26	26	26	26	26	26	26	26	26	26
2000	91.3 ± 3.2	83.64 ± 10.21	537.29 ± 66.92	0.33 ± 0.37	0.04 ± 0.14	0.00 ± 0.00	0.00 ± 0.00	0.29 ± 0.35	0.00 ± 0.00	1.81 ± 2.83
*n*	26	26	26	26	26	26	26	26	26	26

F_1_ generation										

0	86.0 ± 20.1	75.98 ± 17.86	551.21 ± 151.94	0.52 ± 0.83	0.31 ± 0.43	0.00 ± 0.00	0.00 ± 0.00	0.21 ± 0.62	0.00 ± 0.00	1.95 ± 1.77
*n*	22	22	22	21	21	21	21	21	21	21
2000	86.7 ± 19.7	81.57 ± 14.31	557.51 ± 142.72	0.40 ± 0.71	0.19 ± 0.51	0.00 ± 0.00	0.00 ± 0.00	0.21 ± 0.34	0.00 ± 0.00	5.18 ± 15.30
*n*	22	22	22	22	22	22	22	22	22	22

A: without hook, B: banana-like head, C: amorphous, D: folded in midpiece, E: others; HRS: homogenization-resistant spermatids; *n*: number of males per group.

**Table 10:** Litter size and viability index of the F_1_ generation rat pups exposed to SE5-OH during gestation and through lactation of the F_0_ dams.

	Dose (mg/kg/day)							
	0	*n*	200	*n*	1000	*n*	2000	*n*
Number of total offspring at birth								
Males	*#*7.6 ± 2.7	24	7.3 ± 2.5	23	7.4 ± 2.0	22	6.7 ± 1.9	22
Females	7.0 ± 2.0	24	7.3 ± 2.6	23	6.5 ± 1.7	22	7.1 ± 2.3	22
Total	14.6 ± 2.8	24	14.7 ± 1.9	23	14.2 ± 1.8	22	13.8 ± 2.3	22
(M/F)	(183/168)		(169/169)		(162/144)		(147/157)
Number of live offspring at birth								
Males	7.5 ± 2.8	24	7.3 ± 2.6	23	7.2 ± 2.3	22	6.6 ± 2.0	22
Females	6.9 ± 2.0	24	7.2 ± 2.4	23	6.4 ± 2.2	22	7.1 ± 2.3	22
Total	14.4 ± 3.0	24	14.4 ± 1.6	23	13.6 ± 3.3	22	13.8 ± 2.3	22
(M/F)	(179/166)		(167/165)		(159/140)		(146/157)
Number of live offspring on PND 4 before culling								
Males	7.4 ± 2.8	24	7.2 ± 2.6	23	7.1 ± 2.4	22	6.5 ± 1.8	22
Females	6.7 ± 2.0	24	7.1 ± 2.3	23	6.2 ± 2.1	22	7.0 ± 2.3	22
Total	14.1 ± 2.9	24	14.3 ± 1.6	23	13.3 ± 3.5	22	13.5 ± 2.2	22
(M/F)	(178/160)		(166/164)		(156/137)		(144/154)
Number of live offspring on PND 4 after culling								
Males	3.9 ± 0.9	24	4.0 ± 0.2	23	4.0 ± 0.2	21	4.0 ± 0.2	22
Females	3.9 ± 0.4	24	4.0 ± 0.2	23	4.0 ± 0.2	21	4.0 ± 0.3	22
Total	7.8 ± 1.2	24	8.0 ± 0.0	23	8.0 ± 0.0	21	8.0 ± 0.2	22
Number of live offspring on PND 21								
Males	3.9 ± 0.9	24	4.0 ± 0.2	23	4.0 ± 0.4	21	3.9 ± 0.3	22
Females	3.9 ± 0.4	24	4.0 ± 0.2	23	4.0 ± 0.2	21	3.9 ± 0.4	22
Total	7.8 ± 1.2	24	8.0 ± 0.0	23	7.9 ± 0.3	21	7.8 ± 0.5	22
Viability index^A^ (%)								
PND 0	96.86 ± 10.27	24	98.50 ± 4.99	23	95.78 ± 19.81	22	99.62 ± 1.77	22
PND 4	98.23 ± 3.70	24	99.42 ± 1.92	23	93.75 ± 21.38	22	98.58 ± 4.28	22
PND 21	100.00 ± 0.00	24	100.00 ± 0.00	23	98.81 ± 3.76	21	98.30 ± 5.84	22

*#*Mean ± standard deviation; *n*: number of litters; PND: postnatal day; ^A^viability index: PND 0: (number of live offspring born alive/number of offspring born), PND 4: (number of offspring alive on PND 4/number of offspring born alive), PND 21: (number of live weanlings/number of live offspring after culling).

**Table 11:** Litter size and viability index of the F_2_ generation rat pups exposed to SE5-OH during gestation through lactation.

	Dose (mg/kg/day)							
	0	*n*	200	*n*	1000	*n*	2000	*n*
Number of total offspring at birth								
Males	*#*8.0 ± 2.8	18	7.9 ± 2.5	18	6.9 ± 1.8	18	6.9 ± 2.9	20
Females	7.0 ± 2.4	18	6.6 ± 2.7	18	6.6 ± 2.2	18	5.5 ± 2.7	20
Total	15.0 ± 1.9	18	14.4 ± 2.2	18	13.5 ± 2.5	18	12.4 ± 4.2	20
(M/F)	(144/126)		(142/118)		(124/119)		(137/109)
Number of live offspring at birth								
Males	8.0 ± 2.8	18	7.6 ± 2.6	18	6.9 ± 1.8	18	6.6 ± 3.0	20
Females	6.9 ± 2.3	18	6.4 ± 2.5	18	6.6 ± 2.3	18	5.4 ± 2.8	20
Total	14.9 ± 1.9	18	14.0 ± 2.3	18	13.4 ± 2.5	18	11.9 ± 4.5	20
(M/F)	(144/124)		(137/115)		(124/118)		(131/107)
Number of live offspring on PND 4 before culling								
Males	7.8 ± 2.7	18	7.2 ± 3.1	18	6.4 ± 2.1	18	6.4 ± 3.2	19
Females	6.8 ± 2.3	18	6.9 ± 3.0	18	6.3 ± 2.1	18	5.2 ± 3.0	19
Total	14.6 ± 1.8	18	13.2 ± 4.0	18	12.7 ± 2.7	18	11.5 ± 5.0	19
(M/F)	(140/122)		(130/107)		(115/113)		(127/103)
Number of live offspring on PND 4 after culling								
Males	3.9 ± 0.6	18	4.2 ± 0.7	17	3.9 ± 0.5	18	3.9 ± 1.0	19
Females	4.1 ± 0.6	18	3.8 ± 0.7	17	4.0 ± 0.6	18	3.4* ± 1.0	19
Total	8.0 ± 0.0	18	8.0 ± 0.0	17	7.9 ± 0.2	18	7.4* ± 1.3	19
Number of live offspring on PND 21								
Males	3.8 ± 0.6	18	4.2 ± 0.7	17	3.8 ± 0.6	18	3.9 ± 1.0	19
Females	4.1 ± 0.5	18	3.7 ± 0.8	17	4.0 ± 0.6	18	3.4 ± 1.0	19
Total	7.9 ± 0.3	18	7.9 ± 0.2	17	7.8 ± 0.5	18	7.4 ± 1.3	19
Viability index^A^ (%)								
PND 0	99.23 ± 2.23	18	96.92 ± 4.89	18	99.54 ± 1.96	18	96.21 ± 12.29	20
PND 4	97.91 ± 4.15	18	93.61 ± 23.49	18	94.65 ± 12.02	18	94.43 ± 22.30	19
PND 21	98.61 ± 4.04	18	99.26 ± 3.03	17	98.51 ± 4.34	18	100.00 ± 0.00	19

*#*Mean ± standard deviation; *n* = number of litters; PND: postnatal day; ^A^viability index: PND 0: (number of live offspring born alive/number of offspring born), PND 4: (number of offspring alive on PND 4/number of offspring born alive), PND 21: (number of live weanlings/number of live offspring after culling).

## Nomenclature

ANOVA: Analysis of variance
DES: Diethylstilbestrol
ER: Estrogen receptor
GLP: Good laboratory practice
HPMC: Hydroxypropylmethylcellulose
MHW: Ministry of Health and Welfare (Japan)
NOAEL: No-observed-adverse-effect-level.
